# Metabolism of *Seriola lalandi* during Starvation as Revealed by Fatty Acid Analysis and Compound-Specific Analysis of Stable Isotopes within Amino Acids

**DOI:** 10.1371/journal.pone.0170124

**Published:** 2017-01-17

**Authors:** Fernando Barreto-Curiel, Ulfert Focken, Louis R. D’Abramo, María Teresa Viana

**Affiliations:** 1 Facultad de Ciencias Marinas, Universidad Autónoma de Baja California (UABC), Ensenada, México; 2 Thuenen Institute of Fisheries Ecology, Ahrensburg, Germany; 3 Department of Biology, University of Alabama at Birmingham, Birmingham, Alabama, United States of America; 4 Instituto de Investigaciones Oceanologicas, UABC, Ensenada, Baja California, México; National Research Council of Italy, ITALY

## Abstract

Fish starvation is defined as food deprivation for a long period of time, such that physiological processes become confined to basal metabolism. Starvation provides insights in physiological processes without interference from unknown factors in digestion and nutrient absorption occurring in fed state. Juveniles of amberjack *Seriola lalandi* were isotopically equilibrated to a formulated diet for 60 days. One treatment consisted of fish that continued to be fed and fish in the other treatment were not fed for 35 days. The isotopic signatures prior to the beginning of and after the starvation period, for fish in the starvation and control treatments, were analysed for lipid content, fatty acid composition and isotopic analysis of bulk (EA-IRMS) and of amino acids (compound specific isotope analysis, CSIA). There were three replicates for the starvation group. Fatty acid content in muscle and liver tissue before and after starvation was determined to calculate percent change. Results showed that crude lipid was the most used source of energy in most cases; the PUFAs and LC-PUFAs were highly conserved. According to the protein signature in bulk (δ^15^N) and per amino acid (δ^13^C and δ^15^N), in muscle tissue, protein synthesis did not appear to occur substantially during starvation, whereas in liver, increases in δ^13^C and δ^15^N indicate that protein turnover occurred, probably for metabolic routing to energy-yielding processes. As a result, isotopic values of δ^15^N in muscle tissue do not change, whereas CSIA net change occurred in the liver tissue. During the study period of 35 days, muscle protein was largely conserved, being neither replenished from amino acid pools in the plasma and liver nor catabolized.

## Introduction

To understand the efficient use of amino acids by growing fish, several approaches have been investigated to formulate diets for fish in aquaculture [1]. The most common method of estimating requirements is through dose-response curves using a minimum of four treatments with graded levels of high quality protein or amino acids [2]. However, the gap between what is ingested compared to what is retained, as growth remains large, and any reduction in the amount of dietary amino acids will have economic benefits. One approach for improving protein efficiency of dietary formulations is to mimic the proportional composition of amino acids contained in the muscle tissue to assure the presence of all essential amino acids [3]. However, to meet the exact requirements to generate data that can potentially be use to the formulation of efficient feeds, knowledge of the bioavailability of each dietary amino acid to be absorbed and retained is needed [4]. The bioavailability could be indirectly estimated through the determination of the apparent digestibility of the dietary essential amino acids. However, in aquatic organisms, leaching of water-soluble nutrients from both feed and faeces always exists, thereby contributing to inaccuracy in the determination of the amounts of those available amino acids that are actually absorbed. Also, apart from those amino acids retained for anabolic processes (growth, i.e. protein deposition), there is also need to determine those amounts required to meet the demands of metabolic processes. Therefore, the amounts of at least some essential dietary amino acids are commonly underestimated [5].

Earlier studies in our lab used stable isotopes of nitrogen and carbon to estimate the retention level of amino acids from different protein sources, instead of measuring feed ingestion and digestibility [6,7]. The results provided insights into the utilization of protein sources; however, the role of each amino acid in the intermediate metabolism is still unknown. The analysis of stable isotopes in different tissues has been widely used in ecological studies to learn about the nutrient transfer across ecosystem boundaries and to understand trophic relationships and the migration of animals through dietary changes that occur during their life reviewed by Karasov and Martínez del Río [8]. The use of stable isotopes from nitrogen (δ^15^N) and carbon (δ^13^C) contained within their different food sources allows the use of mathematical models to predict how the organic compounds can be differentially retained. Apart from bulk stable isotope ratio analysis (BSIA) where the isotope ratio of bulk samples is measured, the isotope ratios of individual compounds can also be determined. This assay is called compound specific isotope analysis (CSIA) and can be applied to trace individual amino acids to detect their routing [9,10]. If those essential amino acids that are principally retained (growth) can be differentiated from those that have notably additional roles in intermediate metabolism, recommendations about the inclusion of dietary levels of essential amino acids can be improved. Therefore, an appropriate approach to observe how the nutrients are mobilized in the absence of feed input [11], is to distinguish those amino acids used in metabolic processes from those retained by fish under starvation.

In nature, feed deprivation is a usual condition for fish exposed to recurring seasonal fluctuations, such as reproductive condition or availability of prey [8]. Fish have the ability to remain under fasting conditions for short to long periods of time without a severely detrimental effect in physiology [12].

Previous studies have shown that under bulk isotopic analysis, fasting fish exhibit variable rates of enrichment in δ^13^C and δ^15^N relative to diet [13,14,15] suggesting that enrichment might provide some information about the process of nitrogen metabolism to reveal the most limiting amino acids versus those used as energy. According to Lee et al. [16] two alternative processes can occur during starvation and be distinguished by stable isotope analysis: A catabolic model would be based on the loss of high amounts of ^14^N during fasting, resulting in an increase of δ^15^N in all tissues. The second, an anabolic model, proposes that protein synthesis in the liver leads to an increase of δ^15^N in this organ and those organs where protein accretion occurs even under starvation. Because bulk analysis gives the overall information on tissue nitrogen, the use of CSIA under starvation will be helpful to estimate possible differences among amino acids.

Starvation may provide insight into how the nutrients are being mobilized without the confounding interferences that exist in the monitoring of feed ingestion and digestibility. Additionally, it will be possible to estimate how the lipid and protein are mobilized as part of a starvation metabolism to survive [17]. *S*. *lalandi*, also known as yellowtail kingfish, amberjack, or gold-striped, is a pelagic fish that inhabits a subtropical environment and is found in several regions globally [18]. Although *S*. *lalandi* has a comparatively lower metabolism in temperate waters, this species has a higher aerobic scope than that of other fish living in similar temperature ranges [19]. Therefore, the present work was designed with the objective to track certain amino acids and fatty acids under starvation conditions to understand their mobilization from muscle and liver tissue reserves to meet the needs of intermediate metabolism in juveniles of *S*. *lalandi*.

## Materials and Methods

Animal handling was according to our institutional ethical standards (UABC), and approved and supervises by our Institutional Ethical Commission. Juvenile of *Seriola lalandi* were used for the experimental procedure conducted under the certified laboratory for fish nutrition experimental laboratory, and all animal work performed here have been conducted according to the ethics statement from the University (UABC) in accordance to international guidelines. This study was approved and supervised by the ethics commission from the Instituto de Investigaciones Oceanológicas (IIO, UABC).

Samples taken along the experimental procedure and those killed at the end were euthanized by hypothermia in accordance with the University’s policy on health and safety and approval from the supervising commission from the IIO.

*S*. *lalandi* juveniles were provided by Baja Seas SA de CV through the Center for Scientific Research and Higher Education of Ensenada (CICESE). The fish had been reared in a recirculation system under controlled temperature and water quality conditions (22.5 ± 1°C) and fed to apparent satiation three times a day. Upon arrival to the Fish Nutrition Laboratory at the Universidad Autónoma de Baja California, fish (40 g approximately) were placed into four 750 L (500 L volume) tanks that were part of a recirculation system (22.0 ± 1.0°C) and maintained there for 60 additional days at the same feeding rate to achieve a stable isotopic equilibrium (δ^13^C −17.7‰ and δ^15^N 12.57‰) relative to that of the control diet (Alimentos Super SA de CV; Guadalajara Jalisco, Mexico), which contained 42.5% crude protein and 13.1% lipid. The proportional amino acid content of the diet is presented in Table 1. After equilibration, all fish were randomly distributed into each of the four experimental units. In three units, fish were no longer fed, whereas the fourth unit was left as control, continuing to be fed the same diet as previously stated. The control fish were fed daily to apparent satiation at 8:00; 11:00; 14:00 and 17:00 h. The temperature was maintained at 22.8 ±1°C. Three fish from each experimental unit were sampled at time 0, 2, 4, 6, 9, 12, 15, 20, 25, and 35 days. An analysis of fish derived from the control group in addition to the initial samples of fish already equilibrated to the diet was conducted and results were compared with the fish held under starvation for the CSIA or AA analysis.

**Table 1:** Amino acid profile from control diet used to equilibrate the isotopic signature of *S*. *lalandi* and used by the control group.

Amino acid	g/100g
EAA	
HIS	9.5
ARG	6.6
THR	11.7
VAL	4.4
MET	2.1
LYS	4.1
ILE	3.5
LEU	6.1
PHE	2.4
subtotal	50.5
NEAA	
ASP	11.4
SER	6.9
GLU	16.5
GLY	2.3
ALA	2.1
PRO	9.1
CYS	nd
TYR	nd
subtotal	48.2
Others	
TAU	1.3
Total	100

The proximate composition of commercial diet used as control feed; the amounts are given as g/100 of dry feed: crude protein 42.55 ±0.04; crude lipid 13.18 ± 0.07

Samples from muscle and liver tissues were collected and individually tagged; separated and rinsed with distilled water to avoid contamination with other tissues. These samples were then dried at 60°C in a vacuum oven for 36 hours to constant weight (70.0 ± 4.2 and 68.0 ± 1.5% were the moisture contents of the liver and muscle, respectively). Each of the dried samples was individually ground using a mortar and pestle. Both mortar and pestle were thoroughly rinsed between samples. The samples were then weighed and stored in 2 mL Eppendorf tubes at -80°C until analysis. Crude lipid of individual samples was determined according to the Soxhlet procedure [20] whereas the lipid used for fatty acid analysis was extracted according to the methodology described by Folch [21] with some modifications for the latter extraction procedure. Both the liver and muscle tissues, were individually homogenized, and lipids were extracted overnight at 4°C, using dichloromethane-methanol mixture (2: 1, v/v) that contained 1% butylated hydroxytoluene (BHT) to serve as an antioxidant.

The fatty acid methyl esters (FAMEs) of the lipid extracts from muscle and liver tissues were prepared according to Christie [22]. FAMEs were separated in an Agilent Technologies 6880 gas chromatograph equipped with a flame ionization detector (260°C) and a capillary column (DB-23 Agilent; 60 m × 0.25 mm, film thickness 0.25 μm) using hydrogen as the carrier gas. The initial oven temperature was 140°C. One μL of the solution containing the FAMEs was injected splitless and five minutes thereafter the temperature was increased at a rate of 4°C/min to 250°C and then held at that temperature for an additional 10 min. Fatty acids were identified by comparison with the retention times of well-characterized profiles of FAMEs standards (37 Component FAME Mix and PUFA1, Supelco/Sigma-Aldrich). The concentration of each fatty acid was calculated from the corresponding area within the chromatogram using a C_19_ fatty acid as internal standard and the software package Agilent ChemStation (version E.02.00.493).

### Stable isotopes analysis

For the bulk analysis, dried raw muscle and liver tissue samples were analyzed for ^15^N/^14^N and ^13^C/^12^C. Subsamples of 1.25±0.5 mg were weighed using an ultra microbalance XP2U (Metter Toledo^®^ ±0.1μg), placed into tin capsules and sent to the Stable Isotope Facility of the University of California, Davis (USA), for isotopic analysis. According to their laboratory specifications, an elemental analyzer interfaced to a continuous flow isotope ratio mass spectrometer (IRMS) was used. Samples were introduced into the IRMS using helium as the carrier gas, combusted at 1000°C and nitrogen oxides reduced. Thereafter, N_2_ and CO_2_ were separated using a molecular sieve. The laboratory’s internal standards had a standard deviation of 0.3‰. Isotope values are expressed in delta (δ) notation in parts per thousand (‰) relative to VPDB and atmospheric N_2_ as follows:

δX (000)  =  [RXsample − RstandardRstandard]  (1000) 

where, X is ^13^C or ^15^N, R_sample_ and R_standard_ are the ratio of heavy to light isotopes (^15^N/^14^N or ^13^C/^12^C) in the sample and standard, respectively.

For the CSIA for amino acids, dried samples (10±0.5 mg) stored in 0.5 mL Eppendorf tubes were also sent to the Stable Isotope Facility of the University of California, Davis (USA). According to their laboratory specifications samples were treated according to procedures described by Walsh et al. [23]. Samples were hydrolyzed in 6N HCl at 150°C during 70 min under atmosphere of nitrogen, and then dried at 55°C using nitrogen gas flow and re-suspended in 1mL 0.01N HCl. The amino acids were derivatized to methoxy-carbonyl amino acids methyl esters using methyl chloroformate and then injected in split (^13^C) or splitless (^15^N) mode, and separated on an Agilent DB-23 column (30m x 0.25mm ID, 0.25 micron film thickness). Once separated, the amino acids esters were quantitatively converted to CO_2_ and N_2_ in a combustion reactor at 1000°C. For the nitrogen analysis, samples were then dried, whereas CO_2_ was retained in a trapping loop using liquid nitrogen (LN_2_). For the final step of the analysis either N_2_ or CO_2_ entered the IRMS. Neuroleucine was used as internal standard (0.1 mg/mg sample) to help quantification. Final values are reported as δ^13^C (^13^C/^12^C) and δ^15^N (^15^N/^14^N) for each amino acid in ‰, as previously described above for bulk samples.

### Statistical analyses

Student’s t test for independent samples was used to estimate possible significant differences between weight fish, lipid content, fatty acids and the isotopic analysis (in Bulk and per amino acids) between control and starved group. However, when comparison of more than two groups such as the CSIA, among diets and experimental groups, a one-way analysis of variance (ANOVA) was used after testing for homogeneity of variance among repetitions within each treatment. When statistical differences were detected a Tukey *post-hoc* test was used to identify statistical differences between treatments. The differences were considered statistically significant if P < 0.05. All statistical analyses were performed using SigmaStat 3.5 (Systat Software, Inc., Chicago, IL, USA).

## Results

After the adaptation phase of 60 days, the average individual weight of *S*. *lalandi* juveniles was 160.0 ± 10.0 g. After 35 days of starvation, average weight loss was 25.9 ± 9.0% (Table 2) and lipid content in the muscle and liver tissues of fish decreased by 56% and 52%, respectively, as compared to the control group of fish. Average absolute individual wet weight losses for the muscle and liver tissue of fish in the starved treatment were 4.2 ± 0. 4g and 12.7 ± 1.6 g respectively, compared to values of 9.7 ± 0.3 and 26.8 ± 0.6 for muscle and liver, respectively, of fish in the control group (Table 2). The proportional fatty acids compositions of muscle and liver tissues of fed and starved fish are presented in Table 3. According to determined changes in relative content that occurred under starvation, C12:0, C14:0 and C16:0, as well as mono-saturated fatty acids, C16:1n7, C18:1n9 and 18:1n7 were preferentially used in both muscle (except 12:0) and liver tissue, presumably as sources of energy. All were significant changes except for 16:0 in the liver tissue and 18:7n-7 in the muscle tissue. Significant net change decreases of 17.9 mg/ g (18.4%) of muscle tissue and 26.5 mg/g (31.0%) of liver tissue occurred for the C18:2n6 PUFA. For C18:3n3 a relative decrease of 4.4 mg/g (12.0%) in muscle tissue occurred, whereas a significant increase of 57.2 mg/g (525.5%) in liver tissue was observed. The LC-PUFAs such as arachidonic acid (C20:4n6, ARA) resulted in a relative increase of 23.2 mg in each of the muscle (124.8%) and liver (81.4%) tissues. Eicospentaenoic acid (C20:5n3, EPA) decreased in muscle (4.4 mg/g, 7.1%) and significantly decreased in the liver (–22.3g/g, 37.5%), whereas decosahexanoic (C22:6n3, DHA) significantly increased by 128.0 mg/ g (89.7%) in muscle tissue and 86.0 mg /g (50.5%) in liver tissue. In general, standardized amounts of saturated and monounsaturated fatty acids were exhausted, both in muscle and liver tissues, whereas, with some exceptions, the PUFAs and LC-PUFAs were preferentially conserved.

**Table 2:** Total weight loss and crude fat content from muscle and liver tissues of *S*. *lalandi* before and after 35 days under starvation (n = 3) compared to the fed group (control). Initial samples were pool together (n = 6).

Treatment	Weight loss %	Initial		Final	
		Muscle	Liver	Muscle	Liver
Control		9.0 ± 0.4	27.1 ± 0.4	9.7 ± 0.3^a^	26.8 ± 0.6^a^
Starvation	25.9 ± 9.0			4.2 ± 0.4^b^	12.7 ± 1.6^b^

Superscripts letters means significant differences between treatments

**Table 3:** Fatty acids content in muscle and liver tissues (g/ 100g tissue) and net change (g and percentage) of *S*. *lalandi* after 35 days of starvation. Net change per fatty acids was calculated based on the total initial amount and that after 35 days of starvation.

	Muscle				Net change	Liver				Net change
		Control	Starvation	Net change		Initial	Control	Starvation	Net change	
Fatty acid	T 0	35 days	35 days	g	%	time	35 days	35 days	g	%
C12:0	1.29±0.24	1.29±0.23	1.87±0.68	0.58	44.9	2.09±0.86	n/d	0.29±0.05	-1.81	-86.2
C14:0	2.89±0.27^a^	2.85±0.28^a^	0.83±0.20^b^	-2.02	-70.9	2.65±0.46^a^	1.82±0.28^b^	0.88±0.38^c^	-0.94	-51.5
C16:0	19.11±1.34^a^	19.08±0.22^a^	16.76±0.82^b^	-2.32	-12.1	19.47±0.89	19.32±0.22	18.29±2.24	-1.03	-5.3
C16:1n7	4.84±0.61^a^	4.81±0.50^a^	1.42±0.19^b^	-3.39	-70.5	4.14±0.44^a^	3.91±0.50^a^	1.12±0.11^b^	-2.79	-71.4
C18:0	6.85±0.33^b^	7.50±0.32^b^	8.97±0.55^a^	1.47	19.6	8.15±1.73^a^	8.17±0.32^a^	9.12±0.51^b^	-1.15	11.6
C18:1n9	17.53±0.23^a^	17.15±1.18^a^	10.46±2.18^b^	-6.69	-39.0	18.64±1.71^a^	12.94±1.18^b^	8.69±0.60^c^	-4.25	-32.9
C18:1n7	2.18±0.04	2.14±0.42	2.14±0.11	0.00	0	2.20±0.4^a^	2.68±0.42^a^	1.53±0.08^b^	-1.15	-42.8
C18:2n6	10.24±0.39^a^	9.72±0.72^a^	7.93±0.25^b^	-1.79	-18.4	10.74±1.06^a^	8.54±0.72^a^	5.90±1.02^b^	-2.65	-31.0
C18:3n3	2.72±1.52	3.65±2.28	3.21±0.3	-0.44	-12.0	2.21±0.78^b^	1.09±2.28^b^	6.81±2.82^a^	5.72	525.5
C19:0	0.79±0.01	0.77±0.20	0.76±0.11	-0.01	-1.9	0.68±0.06	0.68±0.02	0.72±0.02	0.03	5.1
C18:4n3	0.530.01^a^	0.55±0.06^a^	0.11±0.03^b^	-0.44	-79.8	0.37±0.08^a^	0.25±0.06^a^	0.04±0.05^b^	-0.22	-86.0
C20:4n6	1.87±0.16^b^	1.86±0.12^b^	4.18±0.49^a^	2.32	124.8	2.76±0.24^b^	2.85±0.12^b^	5.17±0.31^a^	2.32	81.4
C20:5n3	6.40±0.1	6.23±0.21	5.79±0.45	-0.44	-7.1	5.14±0.52^b^	5.94±0.21^a^	3.71±0.25^c^	-2.23	-37.5
C22:5n3	2.50±0.31	2.49±0.75	2.92±1.14	0.43	17.1	2.40±0.78	2.41±0.75	3.36±0.42	0.96	39.8
C22:6n3	14.79±1.03^b^	14.23±1.88^b^	26.99±3.88^a^	12.76	89.7	12.60±1.34^c^	16.94±1.88^b^	25.49±1.32^a^	8.56	50.5
Others	5.47	5.67	5.66	-0.01		5.76	10.38	8.89	-1.49	
Total	100	100	100			100	100	100		
ΣSaturate	32.84±1.9	33.38±1	32.19±1.3	-1.19	-3.6	34.95±1.2	36.66±1.5	34.72±1.8	-1.94	-5.3
ΣMUFA´s	25.39±0.7^a^	25.04±1.7^a^	15.00±2.3^b^	-10.04	-40.1	25.93±1.8^a^	22.55±2.3^a^	13.32±1.0^b^	-9.23	-40.9
ΣPUFA´s	34.57±2.6^b^	34.57±2.4^b^	46.53±3.3^a^	11.96	34.6	32.91±0.3^b^	33.47±0.9^b^	47.59±2.4^a^	14.12	42.2
EPA/DHA	0.43±0.0^a^	0.44±0.1^a^	0.21±0.0^b^	-0.22	-51.0	0.41±0.1^a^	0.35±0.1^a^	0.15±0.0^b^	-0.20	-58.5
Σ n3/n6	2.02±0.1^b^	2.05±0.2^b^	2.97±0.2^a^	0.92	44.7	1.54±0.2^b^	2.28±0.8^ab^	2.96±0.4^a^	0.68	29.8

Superscripts letters means significant differences among treatments and initial samples

The isotopic value of δ^13^C and δ^15^N in the control group remained essentially unchanged throughout the experimental procedure.

**Fig 1:**
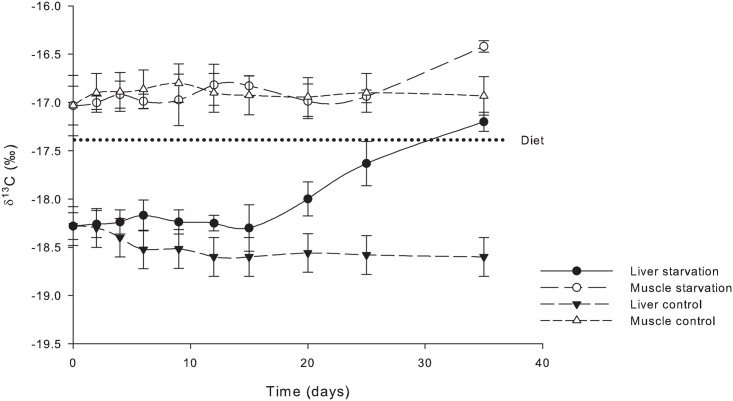
δ^13^C (‰) in bulk muscle and liver tissues of *S*. *lalandi* during 35 days of starvation. Liver starvation, Muscle starvation, Liver control and Muscle control, average and standard variation.

During the first 25 days of starvation, no differences in the isotopic values of δ^13^C of the muscle tissue were observed relative to the control (Fig 1). At day 35, an isotopic enrichment of δ^13^C occurred (0.51‰). In the liver, significant differences in δ^13^C were observed after the first week and remained at that level until day 20 when an additional increase to 1.68‰ occurred by day 35 with respect to the control group.

**Fig 2:**
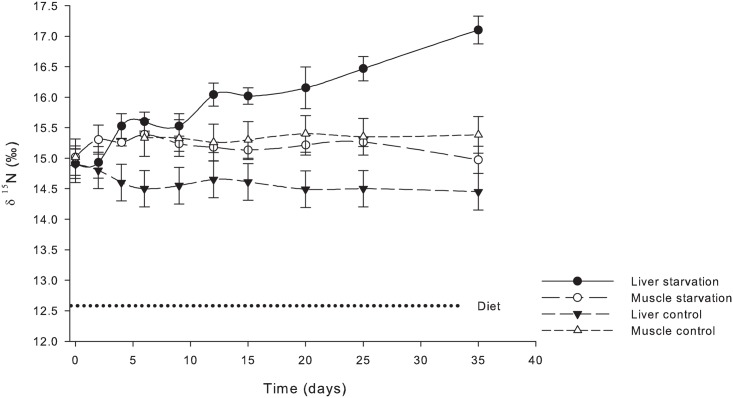
δ^15^N (‰) in bulk muscle and liver tissues of *S*. *lalandi* during 35 days of starvation. Liver starvation, Muscle starvation, Liver control and Muscle control, average and standard variation.

For the nitrogen isotopic value (δ^15^N) in bulk samples, no differences between the control (fed) and starved treatments were observed for muscle tissue (Fig 2). However, within the liver tissue, a significant enrichment occurred among fish in the starvation treatment from day 4 to day 35, when a 2.6‰ enrichment was observed relative to the control group.

**Fig 3:**
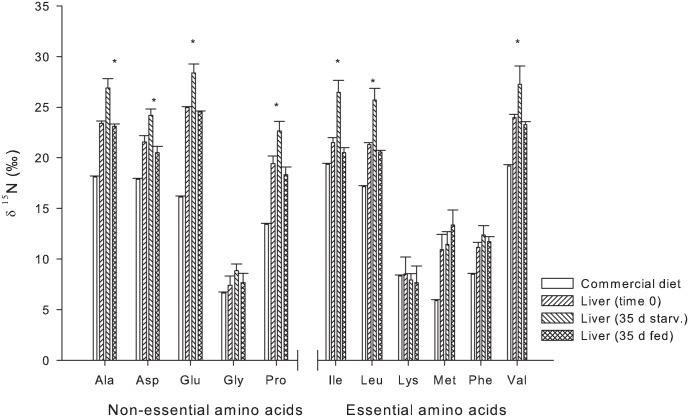
Compound specific δ^15^N of amino acids of of *S*. *lalandi* liver tissue: Commercial diet, Time 0, Liver 35 d starvation and Liver 35 d fed, average and standard deviation. Asterix (*) denotes statistically significant difference between time 0 and 35 days of starvation.

For the compound specific amino acid isotopic values of δ^15^N, no differences were found in the liver tissue of the control group (fed the commercial diet) at time 0 and 35 days. However, the δ^15^N values for fish at time 0 and time 35 days were significantly different from those found in the experimental diet, with the exception of Lys and Gly (Fig 3). Among the essential amino acids (EAA), high enrichment relative to the diet, was observed in the liver for Met (7.5‰), followed by Val (4.1‰), Leu (3.4‰), and Phe (3.2‰) (Table 4). Within the non-essential amino acids (NEAA), the highest enrichment was observed for Glu (8.4‰) in comparison to its equilibrium diet. For muscle tissue, the higher enrichment values were observed in Leu (5.1‰), Ile (4.9‰), Val (4.7‰) and Met (4.2‰), but essentially no enrichment was observed for Lys and Phe. for EAA and for the NEAA, Ala (6.8‰), Pro (5.9‰) and Glu (5.5‰) had the highest enrichment values (Table 4).

**Table 4:** ^15^N enrichment of individual amino acids in liver and muscle tissue of *S*. *lalandi* fed a commercial diet.

Amino acids	Δ δ^15^N (Tissue-Diet)	
	Liver	Muscle
	‰	‰
*Essential amino acids*		
Ile	1.1±0.64	4.9± 0.35
Leu	3.4±0.39	5.1±0.19
Lys	-0.68±0.31	0.05±0.1
Met	7.5±1.66	4.2±1.24
Phe	3.2±0.49	-0.16±0.58
Val	4.1±0.45	4.7±0.34
*Non essential amino acids*		
Ala	4.6±0.88	6.8± 0.27
Asp	2.7±0.71	3.7±0.86
Glu	8.4±0.73	5.5±1.38
Gly	1.0±0.42	1.9±0.05
Pro	4.9±0.82	5.9±0.02

The net change of isotopic value of δ^15^N per amino acid is shown in Table 5, where the initial group as well as the control were considered as the baseline or zero value. The net change observed here after 35 days under starvation shows that the least enrichment in the EAA was observed for lysine in the liver and muscle tissue, Met in the muscle tissue, and Phe for both liver and muscle tissue whereas the most enriched amino acids were Ile, Leu and Val in the liver tissue. For the NEAAs in the liver tissue, Gly was enriched by 2.35‰, followed by Glu, Ala, Asp and Pro (2.8‰, 2.9‰, 3.1‰ and 4.1‰, respectively). Little or no enrichment of these NEAA was observed in the muscle tissue.

**Table 5:** Starvation-induced net changes in ^15^N enrichment of individual amino acids in liver and muscle tissue of *S*. *lalandi* after 35 days of starvation.

	Net change δ^15^N*	
	Liver	Muscle
	‰	‰
*Essential amino acids*		
Ile	5.88±0.18	0.51±0.19
Leu	4.92± 1.17	0.51±0.07
Lys	0.33± 0.33	-1.13±0.40
Met	2.08±0.41	-0.40±0.28
Phe	1.05±0.56	-0.16±0.25
Val	4.29±1.47	0.22±0.30
*Non-essential amino acids*		
Ala	2.89±0.45	0.25±0.35
Asp	3.10±1.28	0.23±0.24
Glu	2.82±0.71	-2.23±0.27
Gly	2.35±0.17	0.51±0.20
Pro	4.12±0.56	0.93±0.19

*Net change = δ^15^N of initial tissue—δ^15^N final tissue

**Fig 4:**
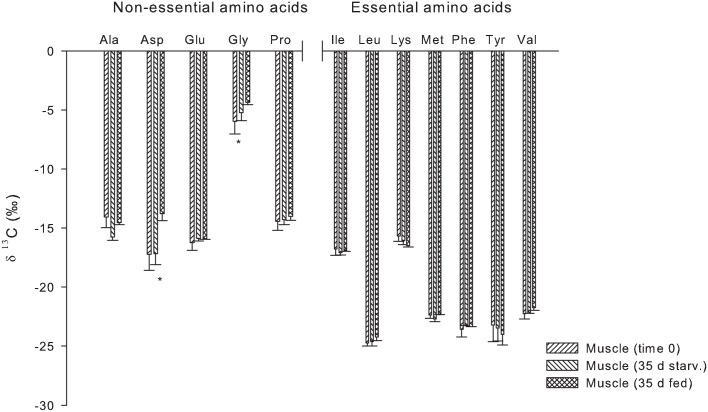
Compound specific δ^13^C of amino acids of *S*. *lalandi* muscle: Time 0, Muscle 35 d starvation and muscle 35 d fed, average and standard deviation. Asterix (*) denotes statistically significant difference between time 0 and 35 days of starvation.

**Fig 5:**
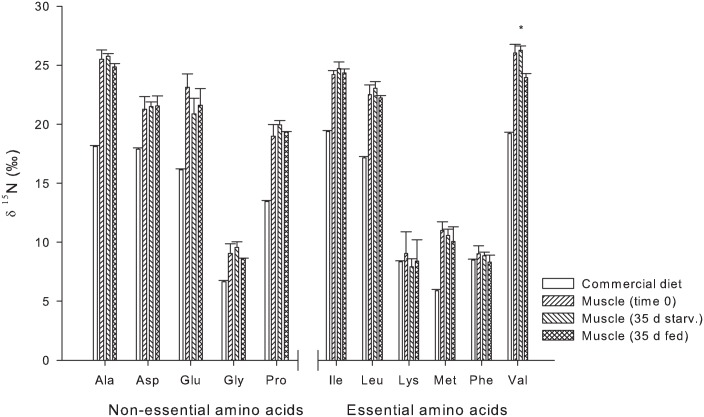
Compound specific δ^15^N of amino acids of *S*. *lalandi* muscle: Commercial diet, Time 0, Muscle 35 d starvation and Muscle 35 d fed, average and standard deviation. Asterix (*) denotes statistically significant difference between time 0 and 35 days of starvation.

After 35 days of starvation, no significant differences (p ≤ 0.05) were observed in δ^13^C and δ^15^N for most of the amino acids in muscle tissue when compared to the initial group (Figs 4 and 5). The only differences observed when compared to the fed group were in δ^13^C Asp and Gly. Whereas, in δ^15^N, only Val was significantly different from that of the fed group.

## Discussion

Fish were presumed to have equilibrated to their diet after being fed the control diet for 60 days, as supported by the observations of Badillo-Zapata et al. [7], before being used in the experimental procedure. Equilibrium was maintained throughout the experimental procedure as confirmed by the bulk analysis for δ^13^C and δ^15^N from samples taken from muscle and liver tissues of fish in the control group at selected times (Figs 1 and 2). These samples were analysed as a whole (without lipid removal); therefore, the δ^13^C measured is derived from AA, carbohydrates and crude lipid, whereas the δ^15^N is derived from protein only.

After 35 days of starvation, *S*. *lalandi* juveniles lost 25.9 ± 9.0% of their wet weight. This percentage loss value was back calculated using the average initial and final weight per tank and represents an estimate because of the variation in individual weight. This weight loss falls within the expected values already reported for carnivorous fish exposed to starvation [11,24]. Thus, standard metabolism was presumably occurring when samples were taken to perform bulk and compound specific isotopic analysis (CSIA) of δ^13^C and δ^15^N from the AAs (fed and unfed), in addition to those collected at the start of the experiment (t 0), and as a series of samples collected for bulk analysis throughout the duration of the experiment. The fish under starvation showed an enrichment of δ^13^C and δ^15^N in bulk samples of the liver tissue, whereas no change was observed in the muscle (Figs 1 and 2). These results are supported by the anabolic model of Lee et al. [16] derived from working with hibernating arctic ground squirrels. During fasting, catabolism is expected to be much higher than anabolism, causing an enrichment of remaining amino acids in ^15^N due to the specificity of glutamate-oxaloacetate dehydrogenase and glutamic dehydrogenase against ^15^N-containing amino acids [25]. However, according to Finn & Dice [26], the proteolysis under starvation occurs in many different tissues through various biochemical processes, whereby the long-term starvation in our study, is activated by chaperone-mediated autophagy. Based on starvation studies using rattlesnakes, McCue [27] postulated the presence of a labile and a non-labile protein pool in the body, with an increasing share of the non-labile protein pool being directed to metabolism during the course of starvation. Fish are poikilothermic animals, with no energy being expended for use in temperature regulation. Therefore, changes under starvation will require a much longer time than those reported for homeotherms [16,26]. In another study using starved poikilotherms [28], no changes in the enrichment of δ^13^C and δ^15^N were observed in bulk analysis of whole-body homogenates of several reptile species.

By applying CSIA of amino acids of different tissues, significant differences were observed between the muscle and liver of the control group and those of the diet. The CSIA for the δ^15^N values of amino acids from liver revealed the largest changes relative to the diet for NEAA, whereas Gly and Lys remained constant. Fantle et al. [29] and McMahon et al. [9] observed that few essential amino acids could be enriched in the heavy isotope without resulting in significant differences with the source. Our investigation revealed that Met was the most enriched AA within the control group as compared to its diet. This observation is surprising as Met is an essential AA. Met is notably required for the synthesis of Tau and *S*. *lalandi* has a high requirement for Tau [30,31]. Usually Tau is directly synthesized from Cys, but, in the absence of Cys, is readily synthesized from Met [32]. In our study Met composed 2.1% of the control diet. The Met requirement for *Seriola* ssp. is listed as 0.8% [1], whereas others report a 1.1% requirement for *S*. *quinqueradiata* [33]. If the Met content in the control diet was higher than required, then Met is probably being used for transamination (e.g. formation of Tau) and deamination and would therefore isotopically behave like a non-essential AA.

Our study did not focus on a comparison of the metabolic routing relative to feeding organisms and different feeding treatments were not included for comparison. However, what can be concluded is that Lys and Gly are the most conserved AA, whereas Glu is the most routed, observations supported by several authors [34,35,36].

Research results derived from studies devoted to the understanding of physiological responses to starvation have been useful for measuring how the energetic reserves are mobilized to provide energy for survival. Crude lipid was among the most used energetic reserves under starvation [37,38]. Most PUFAs are preferentially retained, as indicated by their lack of use as an energy source in fish as also found in warm-blooded animals and freshwater fish [39,40,41,42] and reflected with increase figures. At day 15 in liver tissue and day 25 in muscle tissue, δ^13^C values in the bulk samples show that lipid is being mobilized as an energy source. If our hypothesis is correct, then any protein turnover rate that increased during the period of 15 to 35 days should result in enrichment within these tissues. Values would be noteworthy if the protein synthesis is reduced. According to Rossi et al. [17], under conditions of starvation, glycogen from muscle and liver tissue is the most mobilized source of energy. Protein is mobilized as a source of energy only after glycogen and crude lipid are used. In our study, even as the lipid was already being mobilized from the muscle tissue, no net change of protein synthesis was observed. This is in contrast to the observation by Luo et al. [38] in the channel catfish (*Ictalurus punctatus*) that the muscle crude protein showed a greater decline than muscle crude lipid; and muscle glycogen remained relatively constant during starvation. Theses differences could be attributed to a different physiology between fresh and marine fish and/or different allocation of energy reserves in specific tissues between perciform and siluriform fish. Bender [34] mentioned that during fasting, the protein turnover rate decreases until the starvation is evident. Here, the protein synthesis ceases and only the catabolism of protein can continue, resulting in a loss of protein. When the supply of dietary protein resumes, a balance will be re-established. The results of our study show that the same isotopic signatures for both EAAs and NEAAs are maintained in muscle tissue after 35 days of starvation, indicating a lack of AA synthesis or recycling there.

The total lack of enrichment in muscle suggests that no net change of protein synthesis in the muscle occurred at any time, even during the first period of fasting. Before starvation is apparent, protein synthesis is terminated whereas protein in the liver is being used for the metabolic routing.

The role of Met in the synthesis of Tau and Tau’s role in the production of bile for lipid digestion were previously stated. However, under starvation, no bile is needed, and therefore Met might be used as a glucogenic AA to produce glucose [43,44], which in turn serves as an energy source.

In our study, the AA with the least enrichment was Lys, which therefore can be characterized as the most limiting AA because if Lys were available in excess, no lack of routing or metabolism would have occurred. Earlier published reports [35,36] showed a close relationship between tissue and dietary levels of amino acids in the Pacific bluefin tuna (*Thunnus orientalis*) with Lys enrichment nearly 0‰. In this study, Lys enrichment was lower when compared to the previously reported results of investigators. Lys is also used to synthesize carnitine that mobilizes fatty acids into mitochondria for energy production through β-oxidation [45,46,47].

Phe is considered to be an important indicator of isotopic routing due to its low metabolic participation [48], being highly conserved (an enrichment as low as 0.4 ‰), even across different trophic levels [49,50]. According to earlier published reports, [51,52] the principal role of Phe is the synthesis of neurotransmitters such as dopamine and epinephrine, rather than being utilized in energy producing metabolism [50]. In humans, Phe has been described as a glucogenic AA that can be used to produce glucose [53]. In our study, a Phe enrichment of 1.2 ± 0.5‰ was observed under starvation when compared to that of initial samples. This enrichment probably occurred at the beginning of starvation for use in the synthesis of tyrosine, the immediate precursor to synthesis of these catecholamines [45] or possibly as a glucogenic factor as earlier reported for humans [53].

Val and Ile, both EAAs, were highly enriched in the liver of starved fish, a condition arising from either a high level of utilization or an insufficient dietary level. Both of these AAs are gluconeogenic [47], being catabolised via the citric cycle and utilized for gluconeogenesis. Similar levels of enrichment have been also documented in different organisms such as rotifers and zooplankton from the tropical Atlantic region [35,52,53]. Comparable enrichment patterns were observed for Ala, Asp and Glu, which are considered to be trophic AA [35], and such variations might be influenced by species-specific requirements. Leu is a ketogenic AA whereby a product from its catabolism is CoA that is further converted to acetyl CoA and then to acetoacetate [54,55].

If a minimum of anabolic processes is occurring when food is deprived [11], then Leu could be producing ketone bodies to provide energy for peripheral tissues such as brain and heart [56].

When needed, the NEAAs are being synthesized from different pathways, by either transamination or deamination for gluconeogenesis or lipogenesis [57].

Under conditions of starvation, it appears that the net change in protein turnover rate in the muscle tissue of fish is reduced or terminated, whereas the liver is providing the entire AA required to maintain the metabolic requirements, possibly until severe starvation occurs and all reserves have become metabolised. Accordingly, the lack of change of isotopic values of δ^15^N in muscle tissue combined with CSIA showing a high amino acid turnover reveal that 35 days of food deprivation do not result in severe starvation in *S*. *lalandi*.

When compared to dietary amino acids, fed organisms were significantly enriched, but the magnitude of enrichment was less than that of the starved group. Therefore, we propose that muscle protein is highly conserved during this prolonged, but sub-lethal period of starvation.
